# Combination therapy in a patient with chronic neuronopathic Gaucher disease: a case report

**DOI:** 10.1186/s13256-016-1147-5

**Published:** 2017-01-20

**Authors:** Ferdinando Ceravolo, Michele Grisolia, Simona Sestito, Francesca Falvo, Maria Teresa Moricca, Daniela Concolino

**Affiliations:** Pediatrics Unit, Department of Medical and Surgical Science, University “Magna Graecia”, Catanzaro, Italy

**Keywords:** Neuronopathic Gaucher disease (NGD), Enzyme replacement therapy (ERT), Substrate reduction therapy (SRT), Miglustat, Neurological symptoms, Combination therapy

## Abstract

**Background:**

The variants of neuronopathic Gaucher disease may be viewed as a clinical phenotypic continuum divided into acute and chronic forms. The chronic neuronopathic form of Gaucher disease is characterized by a later onset of neurological symptoms and protracted neurological and visceral involvement. The first-choice treatment for nonneuronopathic Gaucher disease is enzyme replacement therapy with recombinant analogues of the deficient human enzyme glucocerebrosidase. Enzyme replacement therapy has been shown to improve hematological and bone manifestations associated with Gaucher disease, but, as with most proteins, recombinant enzymes cannot cross the blood–brain barrier, which prevents effects on neurological manifestations. Substrate reduction therapy with miglustat (*N*-butyldeoxynojirimycin) inhibits glucosylceramide synthase, which catalyzes the first step in glycosphingolipid synthesis. Because miglustat can cross the blood–brain barrier, it has been suggested that, combined with enzyme replacement therapy, it might be effective in treating neurological symptoms in patients with neuronopathic Gaucher disease.

**Case presentation:**

We report observed effects of combined enzyme replacement therapy and substrate reduction therapy in a 7-year-old Caucasian boy with neuronopathic Gaucher disease who was homozygous for L444P mutations. He had received enzyme replacement therapy from the age of 18 months, and concomitant miglustat treatment was commenced, with dosing according to body surface area uptitrated over 1 month with dietary modifications when he reached the age of 30 months. He experienced mild diarrhea after commencing miglustat therapy, which decreased in frequency/severity over time. His splenomegaly was reduced, and his hematological values and plasma angiotensin-converting enzyme activity normalized. Plasma chitotriosidase also showed substantial and sustained decreases. After 5 years of combination therapy, the patient showed no signs of neurological impairment.

**Conclusions:**

This case supports the concept that oral miglustat in combination with intravenous enzyme replacement therapy may be beneficial in preventing neurological signs in patients with chronic neuronopathic Gaucher disease. The importance of dietary modifications has also been confirmed. Further follow-up studies are needed to better define the therapeutic effect of combined treatment in this Gaucher disease subtype.

## Background

Gaucher disease (GD) is one of the most common lipid storage diseases, with a worldwide incidence of between 1:40,000 and 1:86,000 [1–3], but with a higher frequency among Ashkenazi Jews [4]. GD is caused by a deficiency of acid beta-glucosidase (glucocerebrosidase; GBA) due to autosomal recessive inheritance of mutations in the *GBA* gene (OMIM reference 606463). This enzyme deficiency leads to accumulation of glucocerebroside—the substrate of GBA—in cells of monocyte/macrophage lineage, which then infiltrate the liver, spleen, bone marrow, and other tissues with lipid-laden macrophages. Clinical manifestations of GD include anemia; thrombocytopenia; hepatosplenomegaly; skeletal disease; and, in some patients, neurological involvement [5–8].

GD is usually classified into three phenotypes based on the presence or absence and rate of progression of neurological symptoms. GD type 1 (GD1) is by far the most frequent subtype and does not feature any neurological manifestations [5]. GD type 2 (GD2), the acute form of the disease, features severe neurological involvement and early mortality, usually by the age of 2 years, and GD type 3 (GD3) is characterized by a later onset of neurological symptoms and a more protracted clinical course [8, 9]. It is recommended that GD2 should be described as acute neuronopathic Gaucher disease (NGD) and GD3 as chronic NGD [10]. The major neurological manifestation of chronic NGD is the early development of horizontal supranuclear gaze palsy, which is typically followed by progressive cognitive impairment, myoclonic epilepsy, ataxia, and spasticity [9, 11].

There are two approved treatment modalities for GD. Intravenous enzyme replacement therapy (ERT) is considered as the first-line therapy, comprising chronic biweekly infusions of a recombinant analogue of human enzyme GBA that catalyzes the hydrolysis of glucocerebroside to glucose and ceramide. ERT has been demonstrated to improve hematological parameters (hemoglobin concentrations and platelet counts), organomegaly, and bone involvement [12, 13]. Oral substrate reduction therapy (SRT) with the glucosylceramide synthase inhibitor miglustat (*N*-butyldeoxynojirimycin) is considered as a second-line treatment option for those patients with mild to moderate GD1 for whom intravenous ERT is unsuitable [14, 15].

It must be noted that, because they are proteins, recombinant enzymes do not cross the blood–brain barrier and consequently have been shown to have little effect on neurological disease manifestations in NGD [10, 16]. In contrast, the small-molecule iminosugar miglustat has been shown to have a wide tissue distribution and a partial ability to cross the blood–brain barrier [17, 18]. On the basis of these chemical properties, miglustat was approved for the treatment of progressive neurological manifestations in adult and pediatric patients with the progressive neurodegenerative condition Niemann-Pick disease type C [15]. To date, mixed results have been reported with miglustat in patients with chronic NGD [19–21]. We report a case of a pediatric patient, aged 7 years, with chronic NGD who received early treatment with combination ERT-SRT.

## Case presentation

Our patient was a 7-year-old Italian boy born after an uneventful gestation of normal duration. At the age of 16 months, he presented with a clinically evident enlarged abdomen and was referred for oncological examination. Initial tests revealed anemia, thrombocytopenia, and splenomegaly. A bone marrow biopsy revealed the presence of foam cells, which led to suspicion of lysosomal storage disease. Biochemical testing revealed elevated level of acid phosphatase (47.8 IU/L [normal range 5–7 IU/L]) and chitotriosidase activity (508 nmol/mg protein [normal range 5.9–41.0 nmol/mg protein]), as well as reduced beta-glucosidase activity (2 nmol/mg/protein [normal range 4.5–18.0 nmol/mg/protein]). Molecular analysis showed homozygous L444P mutations in the *GBA* gene (OMIM reference 606463), confirming the diagnosis of chronic NGD.

The patient began ERT at a dosage of 60 U/kg every 2 weeks at the age of 18 months. Thereafter, when the patient reached the age of 30 months, we decided to combine ERT with SRT with miglustat. This clinical decision was approved by our institution’s ethics committee, and informed consent was obtained from the patient’s parents before commencing combination therapy. The dose of miglustat was adjusted according to the patient’s body surface area and was uptitrated during the first 4 weeks with the following scheme: one-third of target dose during weeks 1 and 2, two-thirds of target dose during weeks 3 and 4, and target dose (100 mg three times daily) after 1 month. From 2 weeks before starting miglustat therapy, the patient also followed specific dietary modifications, avoiding high intake of carbohydrate-containing food in single meals, especially foods high in disaccharides, such as sucrose and maltose, to ensure acceptable gastrointestinal tolerability. He experienced mild episodes of diarrhea after commencing miglustat therapy, which decreased in frequency/severity over time.

**Fig. 1:**
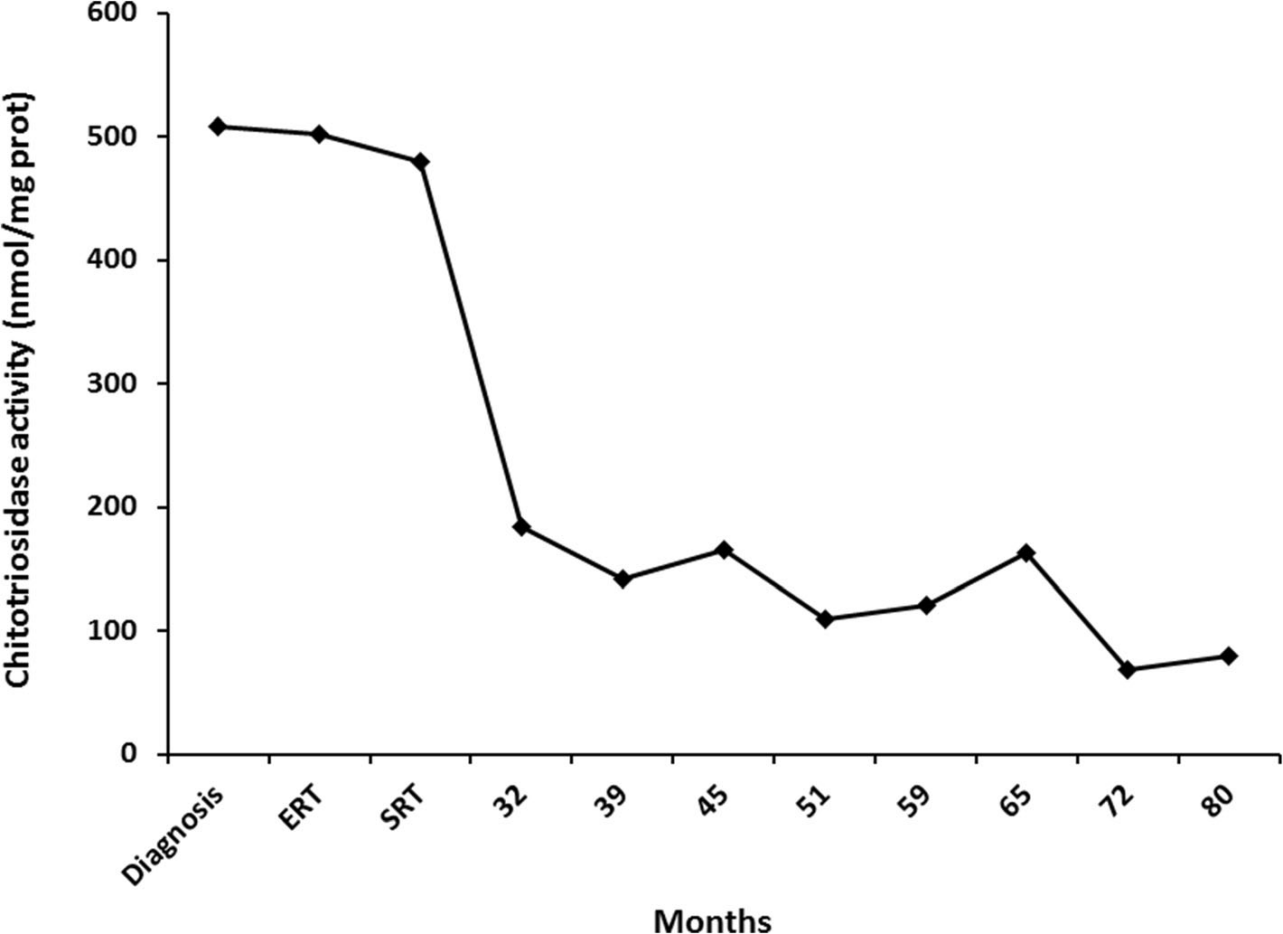
Plasma chitotriosidase over time on treatment. Chitotriosidase values were evaluated approximately every 6 months. These data showed a reduction in plasma values after the start of combination therapy. *ERT* Enzyme replacement therapy, *SRT* Substrate reduction therapy

From the start of ERT/miglustat combination treatment, we observed a reduction in splenomegaly and a gradual normalization of hematological values and plasma angiotensin-converting enzyme activity (Table 1). Plasma chitotriosidase was evaluated at the time of diagnosis and then approximately every 6 months during follow-up. Values showed an initial reduction after the start of ERT, with a further, substantial, and sustained decrease after commencement of miglustat treatment (Fig. 1).

**Table 1:** Hematological and spleen parameters over time on treatment

Age, months (time point)	Hb (g/dl)	PLT (×10^3^ μl)	MCV (fl)	ACE (U/L)	Spleen diameter (cm)
17 (diagnosis)	10.7	185	60.3	–	Enlarged
18 (ERT)	10.5	123	70.6	110	10.4
24 (SRT)	12.2	289	67.5	–	11.0
32	12.3	225	71.1	90	9.5
39	12.7	222	70.6	–	–
45	12.3	246	74.9	90	9.7
51	12.7	256	79.6	–	–
59	12.7	342	74.6	75	8.8
65	12.4	235	75.4	–	–
72	12.8	261	77.4	48	9.5
80	13.5	312	78.7	–	–

Hemoglobin (Hb), platelets (PLT), median corpuscular volume (MCV), angiotensin-converting enzyme (ACE) activity, and echographic measures of spleen diameter were evaluated approximately every 6 months (12 months for ACE and spleen diameter after the first year on therapy). The data showed an improvement in all parameters

The patient has been followed according to recommended guidelines for GD, which specify a complete neurological examination, clinical evaluation of ocular movements, and psychological evaluations every 6–12 months [10]. After 5 years of combination therapy and follow-up, the patient did not show any signs of neurological impairment. As of February 2016, he had not displayed any epileptic crises and had demonstrated clinical performance and cooperation. He showed good muscular tone and trophism, normal reflexes with a slight hyperreflexia in his legs, and a negative Romberg sign. His toe and heel deambulation was normal. In particular, ocular evaluations revealed no evidence of saccadic movement velocity reduction and normal visual evoked potentials. The patient’s auditory brain responses were also normal. In addition, he has not demonstrated any cognitive impairment, and he has regularly attended school with good performance since the age of 5 years.

## Discussion

According to revised recommendations, NGD can be defined as the presence of neurological involvement in a patient with biochemically proven GD [10]. Neuronopathic forms are the rarest variants of GD, although some clusters of high prevalence have been reported, such as that in Norrbotten, Norway, which, unlike nonneuronopathic GD type 1, is not associated with Jewish ancestry [3, 5]. The *GBA* gene is located on chromosome 1q21, and around 400 mutations have been identified that are associated with GD and Parkinson’s disease [22]. Most of these occur in the homozygous state. Our patient carries an L444P/L444P genotype that typically leads to a chronic neurological phenotype [6, 23, 24].

The first clinical signs of neurological involvement in patients with chronic NGD typically appear at a median age of 2 years and worsen with time [6], and L444P-homozygous patients generally show at least one sign of neurological involvement during the early stage or late infantile stage of life [6, 23–25]. In contrast, our patient did not show any neurological symptoms up to his last clinical visit (aged 7 years). This finding is in contrast with our own clinical records of five older patients with the same genotype from a limited geographical area, all of whom received ERT as monotherapy and who presented with neurological impairment during the first 5 years of life.

Positive effects of ERT/SRT combination therapy were first described in 2007 in a 32-year-old patient treated for 2 years with miglustat 200 mg three times daily in addition to ERT (imiglucerase) at a dosage of 60 IU/kg every 2 weeks [19]. The patient showed his first neurological signs at age 22 years. His neurological condition gradually worsened despite ERT, which was increased in dosage to 240 IU/kg every 2 weeks after 5 years. Before the introduction of miglustat, this patient experienced frequent multifocal myoclonic jerks (up to 50 generalized seizures per day) as well as severe dystonia in his arms and legs, which confined him to a wheelchair. After 2 years of combination therapy, there was a marked improvement in his neurological status, including a reduction in seizure frequency to approximately ten per day. In addition, his dystonia improved, and he was no longer confined to a wheelchair.

Other studies have attempted to assess the effectiveness of combination therapy with miglustat plus ERT in NGD, but mixed results have been reported so far. In a 24-month randomized controlled trial with 30 patients with chronic NGD, researchers compared miglustat plus ERT (*n* = 21) with ERT alone (*n* = 9) on the basis of changes in vertical saccadic eye movement (VSEM) velocity from baseline [26]. No significant differences were observed between the treatment groups in VSEM velocity or other secondary neurological parameters, but combination treatment did appear to have a positive effect on pulmonary function and chitotriosidase activity. Although the unexpected positive effect on pulmonary function could justify, by itself, combination treatment with miglustat and ERT, particularly in patients with lung involvement, the neurological findings were not conclusive and left some questions unresolved. First, all patients enrolled in the trial had a reduced VSEM velocity. The lack of asymptomatic patients makes it impossible to determine with certainty the ineffectiveness of combination therapy for the prevention of neurological symptoms. Second, the 24-month observation period was likely too short to identify a therapeutic response in terms of VSEM [26].

Authors of a case series reported findings with combination therapy in three siblings with chronic NGD, all of whom were homozygous for the L444P mutation [27]. The ages of the patients at initiation of combination treatment were 14 years, 10 years and 5 months, respectively. The youngest patient received miglustat in an early stage of the disease, at which point he exhibited only disturbed VSEM and did not show any neurological signs after 3.5 years of therapy. The other patients, who had more established disease and had demonstrated significant neurological impairments from the age of 1.5 years, did not show any neurological improvement. The authors indicated that initiation of combination treatment early in the course of NGD might actually prevent, or at least delay, neurological symptom onset, and that demonstrating neurological improvements in patients with established impairments might be more difficult and/or take longer [27].

Potential beneficial effects of miglustat monotherapy on neurological signs in NGD have also been reported by Accardo and colleagues on the basis of their findings in two sisters with the same genotype (R353G/R353G) who presented with late-onset NGD [21]. Both patients were switched from ERT to miglustat, and both had received barbiturates for control of epileptic seizures [21]. One of the sisters developed oculomotor signs with a VSEM peak velocity reduction (259 degrees/second) that improved (851 degrees/second) after 2 years of therapy with miglustat.

The gastrointestinal tolerability profile of miglustat in our patient was in line with previous published data [28, 29]. Miglustat acts through reversible inhibition of intestinal disaccharidase enzymes (sucrase and maltase) and very mild interference with lactase, which together lead to impaired gastrointestinal carbohydrate absorption and subsequent osmotic diarrhea [28, 30]. In our experience, dietary modifications to reduce the ingested carbohydrate load during drug uptitration in the first month of therapy can improve the gastrointestinal tolerability of miglustat.

## Conclusions

This case supports the concept that the addition of oral miglustat to ongoing intravenous ERT may help prevent neurological deterioration in patients with chronic NGD. The importance of dietary modifications has also been confirmed. Further follow-up studies are needed to better define the therapeutic effect of combined treatment in this GD subtype.

## Abbreviations

ACE: Angiotensin-converting enzyme
ERT: Enzyme replacement therapy
GBA: Glucocerebrosidase
GD: Gaucher disease
GD1, GD2, GD3: Gaucher disease types 1, 2 and 3, respectively
Hb: Hemoglobin
MCV: Median corpuscular volume
NGD: Neuronopathic Gaucher disease
PLT: Platelets
SRT: Substrate reduction therapy
VSEM: Vertical saccadic eye movement
